# Watching Eyes at Home: A Proof-of-Concept Study

**DOI:** 10.3390/bs16040544

**Published:** 2026-04-06

**Authors:** Sabine Windmann

**Affiliations:** The ACT Lab, Department of Psychology, Goethe University Frankfurt, 60323 Frankfurt, Germany; s.windmann@psych.uni-frankfurt.de

**Keywords:** habit building, household recycling, waste sorting, watching eyes, self-awareness, reputation, prosocial

## Abstract

Waste separation in private households remains difficult to promote, particularly in urban contexts, where anonymity limits informal social monitoring. This proof-of-concept study tested, for the first time, self-administration of images of “watching eyes” as an intervention. About 22% of all households living in the district of Riedberg in Frankfurt/Main, Germany, received a letter asking residents to attach eye cues to kitchen and outdoor waste bins to prompt appropriate separation of organic from residual waste. Objective data from weighed collection trucks showed a measurable behavioral effect compared to control conditions, with a 5–8% increase in biowaste volumes. While this study does not allow causal inference because waste was measured only at the group level, it does suggest that, when applied by residents themselves, social nudges might enhance self-awareness about environmentally conscious behavior. Accompanying survey responses displayed ceiling effects, presumably because only highly motivated individuals participated. Importantly, some signs of reactance were also observed, with some participants perceiving the intervention as intrusive and regulatory. Although low-cost and easy to apply, self-administration of watching-eyes cues requires careful communication and attention to psychological reactions to avoid resistance while encouraging the formation and maintenance of target habits in private environments.

## 1. Introduction

Nudging is a behavioral intervention technique that introduces subtle changes in the environment to steer people toward socially desirable behaviors without restricting their behavioral options or incentives (Thaler & Sunstein, 2008; Sunstein, 2014). While many nudges bypass reflective thinking, such as plate size, portion size or product placement, one well-known exception that works through conscious awareness is the use of “watching eyes”, images that are placed close to the location where the target behavior is performed. The simple and powerful effects of watching eyes were first demonstrated by Bateson et al. (2006), who placed images of human eyes above a tea-room honesty box at a university. Contributions for drinks increased during weeks when eye images were displayed compared with control images, suggesting that just activating the concept of being watched can influence cooperative behavior.

Many empirical studies have since shown that displaying images of watching eyes can increase prosocial behavior (Ekström, 2012; Francey & Bergmüller, 2012; Kawamura & Kusumi, 2017; Lv et al., 2024, 2025; van Lange & Manesi, 2023). Experimental studies using economic games show that eye cues, even imagined ones, can enhance altruism and generosity (Lv et al., 2024, 2025), as well as third-party punishment (Li et al., 2021). Contextual factors such as emotional valence of the eyes, perceived realism, and situational cues can modify the effect (Wang et al., 2022). Reviews conclude that the watching-eyes effect is view-specific and might operate through reputation concerns (Jenner & Iredale, 2021; Manesi et al., 2016; van Lange & Manesi, 2023; Wu & Cui, 2020) and other self-referential processing (Conty et al., 2016; Pfattheicher & Keller, 2015). The same seems to hold for reductions in antisocial effects, for which one meta-analysis found small, but nonetheless cost-worthy, effects across domains, while also pointing to contextual moderators (Dear et al., 2019).

If watching eyes work by enhancing self-awareness and concerns about social evaluation, they should also be effective in other behavioral domains where prosocial orientation is essential. Arguably the most urgent is environmentally conscious behavior (van Lange & Rand, 2022). A number of studies have indeed shown that watching eyes help to reduce littering in public spaces (Bateson et al., 2013, 2015; Franzen et al., 2015). Specifically, Bateson et al. (2013), in a field experiment on littering, placed flyers on bicycles and manipulated two things: whether a picture of watching eyes was visible and whether the surrounding area already contained litter (the descriptive norm). They then observed whether people dropped the flyer on the ground or disposed of it properly. The goal was to test whether eye cues increase prosocial behavior in general or simply increase norm conformity. The study found that images of eyes reduced littering, but the effect did not depend on whether the environment signaled a clean or dirty norm. Hence, the watching eyes worked because they had simulated observation of a specific behavior, not more abstract knowledge about social norms.

In another field experiment, Francey and Bergmüller (2012) placed images of eyes or control images (flowers) at bus stops in Geneva. They then observed whether passers-by would remove litter that had been deliberately placed nearby, which counted as contributing to a public good. People were more likely to pick up litter when eye images were present, suggesting that even as subtle, incidental cues placed in public places, watching eyes can increase contributions to public good.

Because watching-eyes cues can influence behavior incidentally and at low cost, they may represent an attractive technique for large-scale behavioral interventions in waste management. Separating organic waste is particularly relevant in this context. If properly separated, biodegradable waste can be processed through composting or anaerobic digestion, producing nutrient-rich compost and renewable energy in the form of biogas. These processes improve soil quality, reduce landfill use, and mitigate greenhouse gas emissions (Ansar et al., 2025). If organic waste is, instead, disposed of in residual trash streams, it will usually be incinerated and, thereby, create CO_2_ emissions. Improving waste separation at the household level, therefore, has significant ecological and economic importance (Alengebawy et al., 2024).

However, organic waste sorting primarily occurs within private households. If the effects of watching-eyes cues rely on reputation formation, as has been suggested (Jenner & Iredale, 2021; Manesi et al., 2016; van Lange & Manesi, 2023; Wu & Cui, 2020), they may be confined to public spaces and locations where individuals can really be observed. By contrast, if the effects are driven by enhanced self-reflection or self-awareness, they might also occur in private environments, at least among individuals who fully endorse the target behavior, but might struggle with reminding or regulating themselves. Under these premises, watching-eyes cues might help to develop the desired behavior from a consciously controlled action into an automated habit, similar to what has been proposed for implementation intentions (Gollwitzer, 1999; Gollwitzer & Sheeran, 2006).

**Figure 1:**
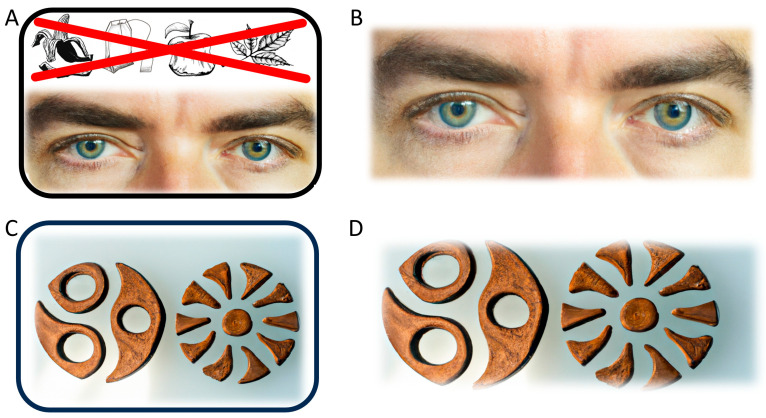
Images used in this study (AI generated): (**A**) the sticker used in the intervention condition of the present study, designed in accord with Lotti et al. (2023); (**B**) the 20 × 10 cm long tag used in the intervention study to be placed on the outside trash bin; and (**C**) sticker used for control condition (KG), and (**D**) tag for the outside bin used in the control condition (KG).

The present study used images of watching eyes as nudges in private households. The cues were designed to discourage the disposal of organic waste in the residual trash bin. Participation in this study was entirely anonymous to reduce reactive effects on measured behavior (the Hawthorn effect), as well as for ethical reasons. Households were randomly selected in each of three districts of Frankfurt Riedberg. They were each provided an envelope containing a sticker and a door tag depicting watching eyes (male eyes with a neutral expression, see Figure 1) via their mailboxes. Recipients were instructed through a flyer (see Supplementary Material) to place these materials either on or near the residual trash bin in their kitchen and/or on the outside waste bin for a period of two weeks.

This intervention represents, to our knowledge, the first test of self-administration of a watching-eyes cue in a naturalistic field setting. Rather than being installed by authorities or institutional administrations, the cues were placed by residents themselves, either on or near the residual trash bin in their kitchen or on the external waste bin for a period of two weeks. This design allows examination of whether watching eyes as strong social cues remain effective when voluntarily implemented by the observed subjects themselves, thereby potentially strengthening self-awareness and self-regulation processes (Conty et al., 2016; Pfattheicher & Keller, 2015).

The aim of the intervention was to reduce the proportion of organic waste disposed of in the residual trash stream. As the primary dependent measure, the amount of organic waste produced by all households together was recorded and weighed by the municipal waste management company, the Frankfurter Entsorgungs- und Service GmbH (FES). The weighing included wastebins not only of households that had participated, but of all households in the district, so that the results would probably reflect an underestimation of the intervention effects.

In addition to the objective data collection, the letter included an invitation to participate in a brief online survey to gather supplementary data on subjects’ waste-sorting habits and self-reported participation compliance. This combination of behavioral and self-report measures makes it possible to assess not only whether the self-administered watching-eyes cue produced measurable changes in waste-sorting behavior, but also how participants perceived the intervention. Such a combination is important because behavioral change and subjective evaluation do not always go in the same direction. An intervention may produce small measurable improvements while, at the same time, evoking ambivalent feelings. Understanding both aspects is, therefore, essential for evaluating practical feasibility and long-term acceptance.

Hypotheses were that, relative to before-treatment measurements, the weight of the organic waste would be higher in groups that were given watching eyes compared to control groups, who had received either a dummy image or no image. For the survey, reports of habitual organic waste-separation habits were expected to be rated higher in the treatment groups compared to the control groups.

## 2. Materials and Methods

Twenty-four student assistants distributed letters containing the sticker and the tag to approximately 2000 households as part of a project seminar. In line with the findings of Lotti et al. (2023), the stickers featured a graphic illustration of typical organic waste items (apple core, banana peel, etc.) placed above the watching eyes, crossed out with red lines to signal improper disposal in the residual waste (non-recyclable trash) bin (see Figure 1). The image was generated using artificial intelligence (AI) tools that were freely available at the time. In addition, groups were given a cardboard tag for the outside bin that contained the same image (without the organic waste items). The letter, in the form of a flyer, explained the purpose of the campaign and the instructions (see Supplementary Material). Approximately one-third of the households (Riedberg West, HC group) received only minimal instructions under the heading “Mindful waste separation”; a second third of the households (Riedberg North, HC+ group) was additionally informed that more than 30% of residual waste from Frankfurt households was actually organic waste and that this fraction was missing as an energy source in biogas plants. The third group (Riedberg South, active control group, KG) served to control for placebo effects and, to that end, received a sticker and pendant with two Yin/Yang-like images (AI generated, see Figure 1), together with the instruction to apply these images as a reminder to be mindful of their own health and well-being. Instructions are given in full in the Supplementary Material.

This study was preregistered at https://aspredicted.org/d3nr2.pdf on 1 December 2023. We preregistered that the weight of the organic waste at the last collection time point before the intervention (1 December 2023) would be compared with the first time point after the intervention (pre–post comparison) among the three groups, HC and HC+ versus KG. Weighing was specific to the three groups, not at the level of individual households. For that reason, only one measure per group was available, so that we obtained only descriptive (no inferential) statistics. We, therefore, consider this study a feasibility study, rather than a test of treatment effects in the strict sense.

In addition to the preregistered design, a passive control group (from the district Frankfurt Nieder-Eschbach, group P) was added retrospectively, in order to better assess the robustness of the observed effects. This addition appeared informative because the collection schedule in the active control group (KG) turned out to be shifted by one week, relative to that of the two treatment groups (HC and HC+), a circumstance that was not known at the time of the intervention. The shift may have introduced temporal noise or seasonal fluctuations to the data. By including a passive control group operating on an unchanged schedule, we secured the stability and interpretability of the effects found in the original comparison.

Note that all intervening events were the same in all four groups, including weather conditions, which were typical for the season, albeit quite warm in the last week of the sampling epoch. November of 2022 in Frankfurt Riedberg was characterized by typical, relatively mild, and frequently rainy conditions, with average temperatures ranging from 35 °F to 48 °F (2 °C to 9 °C). This period often coincides with the peak of autumn leaf fall, necessitating high-volume green waste disposal. In December, temperatures dropped, at first, to near-freezing conditions of 35 °F to 48 °F (2 °C to 9 °C), then rose significantly around the 20th, with daily highs reaching 50 °F to 55 °F (10 °C to 13 °C) during Christmas week. The weight measurements ended before New Year’s celebrations in all groups.

## 3. Results

**Figure 2:**
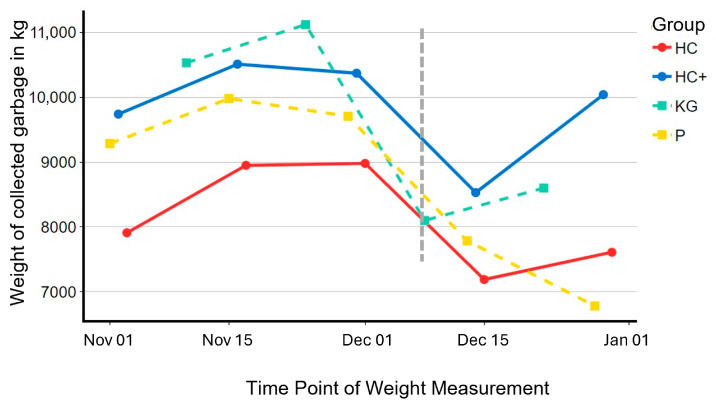
Weight of organic waste collected in treatment groups (HC and HC+), control group (KG) and passive control group (P). The dashed grey line indicates the date of the intervention.

Results of the organic waste weighing are presented in Figure 2. Across the observation period, all four groups showed high variation in the amount of organic waste collected. These fluctuations are likely explained by weather conditions and arising garden waste, such as accumulated leaves and green cuttings. Around the time of the intervention, the weight of organic waste declined in all groups, which is consistent with typical seasonal patterns in November and December. Specific local events, holidays, or festivities that may have affected the variation are not known for the chosen period.

The key issue for evaluating the intervention is whether the weight decline was less pronounced in the treatment groups (HC and HC+) than in the control groups (KG and P). If the decrease was smaller in the treatment groups, this would suggest that the watching eyes had helped maintain or increase the correct disposal of organic waste.

**Figure 3:**
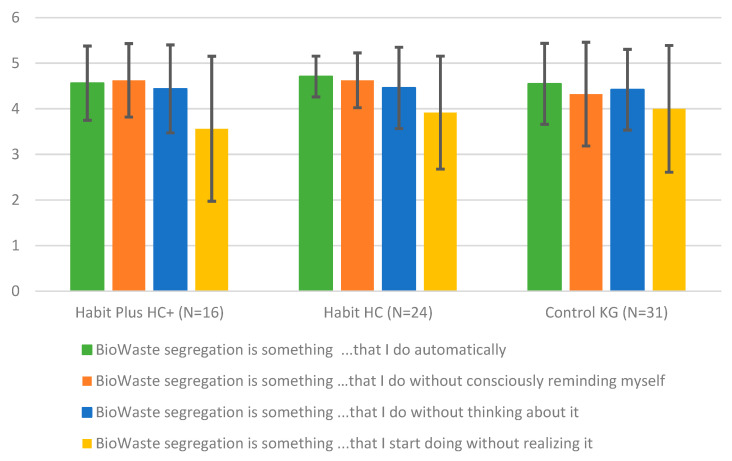
Online survey on the self-rated degree of automaticity of organic waste-separation behavior in the two treatment groups (Habit = HC and Habit Plus = HC+) and control group.

As shown in Figure 3, the reduction from before to after the treatment was smaller in the two treatment groups compared to controls. Specifically, organic waste weight decreased by 19.7% in HC and 18.8% in HC+, compared with a 27.2% decrease in the active control group (KG) and a 22.0% decrease in the later-added passive control group (P).

This pattern suggests a relative advantage for the treatment conditions. In the preregistered design, the difference corresponds to an improvement of roughly 8% in correct organic waste separation. When the passive control group is included in the comparison, the effect is reduced to about 5%. It should be noted, however, that the passive control area (Karbach) was not fully comparable to the Riedberg district in terms of demographic characteristics, and was incorporated only after the initial design had been specified.

In summary, the results show a consistent tendency in favor of the intervention, despite the fact that less than every third household received an invitation letter, leading to underestimation of the true effect size. In any case, the observed differences require further study to determine their statistical reliability and practical relevance. Variability in household routines, seasonal influences, and structural differences between residential areas may have contributed to fluctuations in waste volumes and limit firm conclusions about robustness.

In addition to the objective weighing data, which capture actual disposal behavior, an online survey assessed habitual organic waste separation. The questionnaire was a four-item measure on habitual behavior, previously validated in other behavioral contexts (Gardner et al., 2012), to be answered on a five-point Likert scale ranging from “rarely or quite rarely” to “often or very often”. One control question “Please simply check rarely or very rarely here” was added to screen for careless responding. In addition, participants were asked whether they had, in fact, applied the sticker/tag or were about to do so. Another question checked whether they had understood what was asked of them and had an organic waste bin at their disposal. A total of 137 individuals participated in the survey; 98 provided complete responding. Of these, 71 (50 female) answered the control item correctly and were included in the final analysis. The self-report data complement the behavioral findings by offering insight into participants’ compliance with study instructions and their reported organic waste-sorting habits. Results are presented in Figure 3; German version of the items is provided in the Supplemental Material.

Overall, the findings suggest that participants in the two treatment groups (HC and HC+) were somewhat more likely than those in the active control group to describe organic waste separation as a habitual or automatic behavior. However, interpretation is limited by a clear ceiling effect: many respondents selected the highest possible value (a rating of 5) on the response scale. This restriction reduces variability and makes it difficult to detect meaningful differences between groups. At the same time, it indicates that survey participants were generally highly committed to proper waste sorting from the outset, indicating a high selection bias in the sample of this study.

In addition, one survey item (yellow-shaded in Figure 3) appeared to be slightly more difficult than the others or may have caused some irritation. The wording may have been unclear, possibly due to misunderstanding of the item in German. The original “… I start doing without realizing it” was translated literally into “… das ich anfange zu tun, bevor ich es realisiere”. This phrasing may have felt a bit uncommon because the word “realizing” appears slightly unusual in this context in German (but not weird or incorrect). As a result, responses to this item may contain additional measurement error. Future studies should revise and pretest the translation to ensure that its meaning is clear and interpreted consistently across respondents.

The qualitative feedback also provides useful context. Approximately a dozen open-ended comments were submitted. These fell into three roughly equal categories: (1) expressions of approval for the intervention, (2) statements emphasizing that respondents already separated waste properly regardless of additional cues, and (3) criticism of the specific materials used (e.g., that the stickers were not aesthetically appealing; such comments came primarily from the control group).

Beyond the survey, several residents who chose not to participate contacted the principal investigator by email or post. Three individuals returned the materials, expressing strong disapproval because they perceived the watching-eyes motif as an attempt to regulate or monitor their behavior. One note included the statement: “Der größte Lump im ganzen Land, das ist und bleibt der Denunziant” (“The greatest scoundrel in the whole country is, and always will be, the informer”). These reactions suggest that some residents view waste management strictly as a matter of private choice. They also illustrate the strength of the symbolic association between eye imagery and surveillance. Notably, this perception emerged despite voluntary and anonymous participation and the intention to frame the cue as supportive, rather than coercive.

## 4. Discussion

In the present study, a behavioral effect of the watching-eyes cues was demonstrated when these nudges were self-administered to improve waste recycling in private households. Although we cannot evaluate the statistical reliability of the effect, this study provides proof of concept, given that two treatment conditions show the expected decline in waste weight compared to the two control conditions. By contrast, differences in the accompanying information (HC versus HC+), regarding the value of organic waste for composting facilities and biogas plants, did not appear to influence this pattern. This means that the additional informational framing about environmental and economic benefits did not strengthen the behavioral effect that originated from the visual cue itself.

This might suggest that the watching eyes stimulus may operate through mechanisms that are relatively independent of explicit validation. In other words, the visual cue alone may be sufficient to activate psychological processes related to being observed, even when no detailed explanation is provided. This point is relevant for practical implementation, because it means that complex informational campaigns may not always be necessary, or may not be sufficiently efficient to trigger behavioral changes, one of the greatest advantages of nudging. On the other hand, the lack of difference between the two treatment conditions may be related to the ceiling effect observed in the survey. Participants may already have been highly motivated or well informed, such that the information provided in HC+ was unable to further improve their already exemplary waste-sorting behavior.

Consistent with our interpretation of the objective differences in the weight measures that were independent of explicit explanations, several other studies indicate that the effect of eye cues emerges even in the absence of detailed normative explanations or material incentives. For example, Bateson et al. (2006) showed that simple images of eyes increased honesty in a real-world payment setting without any accompanying persuasive message. Similarly, Francey and Bergmüller (2012) reported increased contributions to a clean environment when eye images were displayed, although no additional informational framing was provided. In a different domain, Nettle et al. (2013) demonstrated reduced bicycle theft following the placement of eye-themed signage, again without elaborate explanation of monitoring. In all these cases, watching eyes operated at the level of subjective experience without addressing explicit arguments.

Our findings are encouraging at the behavioral level, but, at the level of subjective experience, they are limited in clarifying the mediating processes further. There is no clear consensus in the literature regarding why and under which conditions watching eyes are effective (Kawamura & Kusumi, 2017). One central explanation refers to self-awareness and self-reflection (Conty et al., 2016; Pfattheicher & Keller, 2015). According to this account, eye images increase self-focused attention and heighten awareness of personal standards. When attention is directed toward the self, discrepancies between actual behavior and internal norms become more salient, which can motivate self-controlled corrective action. This reasoning matches with control theoretical models of self-regulation (Berkman et al., 2017; Carver & Scheier, 1998; Gollwitzer, 1999) and implies that eye cues may strengthen internal monitoring and control processes. We had, therefore, asked participants in the survey how automated their waste sorting felt after the intervention, using the short version of a prominent questionnaire (Gardner et al., 2012). Since the responses showed ceiling effects, we have no strong evidence of differences between the watching-eyes cue intervention and the control groups to clearly corroborate this mechanism. It should be noted, however, that even among motivated individuals who already show adequate waste-separation behavior, waste-separation reminders can produce additional positive effects, like increases in wellbeing (Mao et al., 2025).

Without additional research, the question of whether habit forming by increasing self-awareness is independent of habit formation by means of reputation formation must remain unclear. Conty et al. (2016) have provided a review about self-referential processing induced by watching eyes and argue that eye cues are effective when they activate self-related representations. The presence of eyes enhances the integration of social perception with one’s self-concept, particularly in contexts that already encourage reflection. Likewise, Pfattheicher and Keller (2015) believe that watching eyes increase the subjective sense of being observed, which raises “public self-awareness”. Their data support a mediation model, in which perceived observability leads to heightened public self-awareness, which, in turn, predicts greater adherence to social norms. These frameworks connect reputational concerns with self-awareness processes and suggest that both may operate together, rather than independently. Future studies should examine what mechanism operates best in private environments.

In the present field context, the watching eyes intervention produced a small improvement in organic waste separation, as assessed by generated weight of waste. It should be noted that the magnitude of the observed difference may actually be an underestimation of the effect because a considerable number of households whose waste contributed to the aggregated weight data did not receive an invitation to participate. Their inclusion in the measurement inevitably reduced potential differences between conditions. In addition, shared containers and overlapping disposal routines increased variability and reduced measurement precision. These structural features limit strong causal claims.

### Limitations and Management Implications

Due to the limited variance in the weight data, we are unable to conduct inferential statistical tests, which clearly makes this study a pilot. Furthermore, the survey responses show ceiling effects, likely due to selection bias, meaning that the sample is probably not representative for households of a large Western city. However, the data provide some qualitative feedback indicating dissociate reactions to the intervention. Some individuals were enthusiastic about our study design, while others apparently felt outright offended. Presumably, when individuals perceive that they freely choose to use the sticker, it may strengthen their sense of autonomy and internal responsibility. In contrast, if they perceive social pressure or covert monitoring, the same stimulus condition may threaten their sense of autonomy and provoke resistance. This distinction is especially relevant in private domains, such as household waste management, where individuals expect a high degree of personal freedom. Participants who already valued waste separation tended to interpret the image as a reminder that helped to strengthen or support existing habits. For some others, particularly those who sent the letter back, the same image was perceived as intrusive or moralizing. For the latter, although they were a small minority, the intervention proved inappropriate. However, their reaction is consistent with classical psychological reactance theory (Brehm, 1966; Steindl et al., 2015), as well as with the idea that public self-awareness can produce discomfort when individuals feel judged. The perceived legitimacy of the intervention, therefore, appears crucial, and needs to be considered during planning of any watching eyes intervention, even under self-administration.

This points to several other features of the current study that practicians might want to consider. The accompanying letter should build trust by clearly explaining the intended mechanism and by emphasizing the voluntary nature of the intervention to improve self-awareness and self-control, if needed or desired. The design of the stimulus could be changed to reflect a friendly, benevolent look rather than a controlling or moralizing one (Dear et al., 2019; Lau et al., 2023). In addition, it could be designed as a positive cue to encourage the desired organic waste-sorting behavior, rather than a discouraging, stern gaze that appears to signal condemnation of inappropriate disposal of organic waste in the residual trash. Behavioral measures should be collected at the level of individual households where feasible and considered ethical, although we do not see an easy way of implementing this without breach of privacy. Finally, longer follow-up periods are necessary to assess persistence, as formation of stable habits typically requires repeated performance over extended time (Gardner et al., 2012; Wood & Rünger, 2016). What needs to be checked particularly is whether watching-eyes cues lose their efficiency after some time due to habituation (in the sense of extinction) and need to be renewed or replaced by other nudges to support the formation and maintenance of the target habits (Jenner & Iredale, 2021).

The self-administration of watching-eyes cues is a distinctive feature of this study. This increases real-world applicability while highlighting personal responsibility. For some people, self-placement may strengthen ownership and internalization; for others, it may still signal normative control. In addition, variability in placement, visibility, and exposure duration, as well as participant and stimulus characteristics (Lau et al., 2023), may moderate cue effectiveness. Systematic comparisons between self-administered, externally installed cues and those administered by experts in behavioral training (coaches, therapists, scientists) could examine whether the observed effects generalize across modes of implementation or depend on the specific way the cue is introduced into everyday environments. Such research could also examine the boundary conditions of the treatment, for example by testing different visual designs, different emotional expressions of the eyes, or different durations of exposure (Lau et al., 2023; van Lange & Rand, 2022). It would also be useful to investigate whether repeated short interventions over time are more effective than one continuous exposure. Finally, combining watching eyes with other behavioral strategies, such as feedback on waste amounts or descriptive norm information, could help to determine whether additive or interactive effects occur. A more differentiated understanding of these mechanisms would strengthen both theoretical accounts and practical applications.

## Data Availability

The original contributions presented in this study are included in the article/Supplementary Material. Further inquiries can be directed to the corresponding author.

## Supplementary Materials

The following supporting information can be downloaded at: https://www.mdpi.com/article/10.3390/bs16040544/s1, Figures S1–S4: Flyers delivered in the Experimental Conditions and Translations; Figures S5 and S6: Flyers delivered in the Control Condition and Translation. Figure S7: Screenshot of the habit questionnaire (original).

## Abbreviations

The following abbreviations are used in this manuscript:

HC	Habit Creation
HC+	Habit Creation Plus
KG	Control
P	Passive Control
