# Supplementary material for: Watching Eyes at Home: A Proof-of-Concept Study
Source: Behav Sci (Basel). 2026 Apr 6;16(4):544. doi: 10.3390/bs16040544 (PMC13113408; doi:10.3390/bs16040544)
Supplement: Supplementary file 1 [file behavsci-16-00544-s001.zip › behavsci-4179241-supplementary.pdf]

Supplementary Material: Watching Eyes at Home (by Sabine Windmann)

Figure	Caption
S1	Flyer delivered to Experimental Condition HC+ (Original)
S2	Flyer delivered to Experimental Condition HC+ (Translation to English)
S3	Flyer delivered to Experimental Condition HC (Original)
S4	Flyer delivered to Experimental Condition HC (Translation to English)
S5	Flyer delivered to Control Condition KG (Original)
S6	Flyer delivered to Control Condition KG (Translation to English)
S7	Screenshot of the short habit questionnaire (Original)

Flyer (Original): Treatment Group HC+

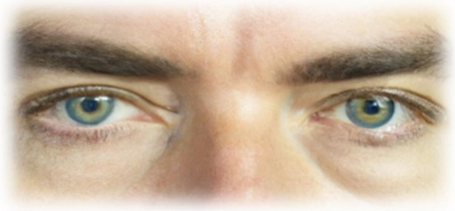

„Mindful“ Mülltrennung

Eine Psychologische Intervention

Sehr geehrte Anwohnerin, sehr geehrter Anwohner,

Wussten Sie, dass **mehr als 30% des Restmülls** der Frankfurter Haushalte eigentlich Biomüll ist?

Dieser wertvolle Biomüll **fehlt** dadurch in der Biogasanlage für die Energiegewinnung!

Hier ist, was Sie tun können:

Sammeln Sie Biomüll durch „**Mindful**“ **Mülltrennung**:

- ✓ Kleben Sie die Augen auf den Deckel Ihrer **Rest**mülltonne (z.B. in der Küche) oder gut sichtbar in die Nähe (z.B. Arbeitsplatte, Schublade, Schrank). Die Augen sollen ihre **Achtsamkeit** erhöhen: ***Kein Biomüll*** **in die Restmülltonne!**
- ✓ Befestigen Sie den Anhänger für zwei Wochen **draußen an der schwarzen Rest**mülltonne (z.B. am Griff des Deckels), in der Sie üblicherweise Ihren Restmüll entsorgen. So zeigen Sie nach außen, dass Sie bei der Aktion mitmachen.
- ✓ Nehmen Sie online an unserer Kurzumfrage teil.

Weitere Information: Die Teilnahme an dem Projekt ist vollkommen **freiwillig**. Aufkleber und Anhänger können Sie auf jeden Fall kostenfrei behalten. Die Befragung dauert etwa **2 Minuten** und Sie können einen von drei Gutscheinen über 100 Euro gewinnen! Weitere Informationen erhalten Sie entweder mit dem Barcode oder unter dem Link:

https:/tinygu.mindful

[Barcode hier]

Projektleitung:

Prof. Dr. Sabine Windmann
Psychologisches Institut Goethe Universität Frankfurt
s.windmann@psych.uni-frankfurt.de | Tel. 069798 35313

Flyer (Translation): Treatment Group HC+

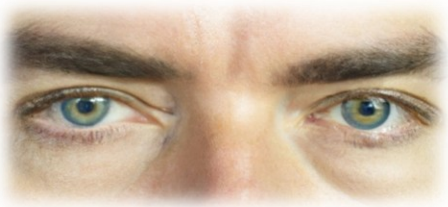

„Mindful“ Waste Separation

A Psychological Intervention

Dear resident,

Did you know that **more than 30% of Frankfurt households' residual waste** is actually organic waste?

This valuable organic waste is therefore **missing** in the biogas plant for energy production!

Here is what you can do:

Collect organic waste through “**mindful” waste separation**:

- ✓ Stick the eyes onto the lid of your **residual** waste bin (e.g., in the kitchen) or place them clearly visible nearby (e.g., countertop, drawer, cabinet). The eyes are intended to increase your **mindfulness**: ***No organic waste*** in the **residual waste bin**!
- ✓ Attach the tag to your **black residual waste** bin **outdoors for two weeks** (e.g., on the handle of the lid), where you usually dispose of your residual waste. This way, you show outwardly that you are participating in the campaign.
- ✓ Take part in our short online survey.

Further information: Participation in the project is completely voluntary. You may keep the stickers and tags free of charge in any case. The survey takes approximately 2 minutes, and you can win one of three vouchers worth €100! Further information is available via the barcode or at the link:

https:/tinygu.mindful

Projekt Director:

Prof. Dr. Sabine Windmann
Psychologisches Institut Goethe Universität Frankfurt
s.windmann@psych.uni-frankfurt.de | Tel. 069798 35313

[Barcode here]

Flyer (Original): Treatment Group HC

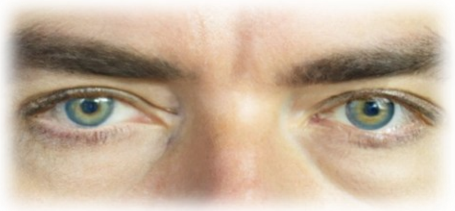

„Mindful“ Mülltrennung

Eine Psychologische Intervention

Sehr geehrte Anwohnerin, sehr geehrter Anwohner,

wir führen eine Aktion durch in Kalbach-Riedberg.

Sie sind per Zufall ausgewählt worden, in einer Gruppe des Projekts mitwirken zu können. In dieser Gruppe möchten wir die **Bio-Mülltrennung verbessern**. Wir mächten Sie ermuntern, **achtsam** mit dem Biomüll umzugehen.

Wir bitten Sie zusätzlich, an einer Befragung teilzunehmen, die genau **diesen Fokus** hat (Trennung von Biomüll im Haushalt).

Hier ist, was Sie tun können:

Sammeln Sie Biomüll durch **„Mindful“ Mülltrennung**:

- ✓ Kleben Sie die Augen auf den Deckel Ihrer **Rest**mülltonne (z.B. in der Küche) oder gut sichtbar in die Nähe (z.B. Arbeitsplatte, Schublade, Schrank). Die Augen sollen ihre **Achtsamkeit** erhöhen: **Kein Biomüll in die Restmülltonne!**
- ✓ Befestigen Sie den Anhänger **für 2 Wochen draußen an der schwarzen Rest**mülltonne (z.B. am Griff des Deckels), in der Sie üblicherweise Ihren Restmüll entsorgen.
- ✓ Nehmen Sie online an unserer Kurzumfrage teil.

Weitere Information: Die Teilnahme an dem Projekt ist vollkommen **freiwillig**. Aufkleber und Anhänger können Sie auf jeden Fall kostenfrei behalten. Die Befragung dauert etwa **2 Minuten** und Sie können einen von drei Gutscheinen über 100 Euro gewinnen! Weitere Informationen erhalten Sie entweder mit dem Barcode oder unter dem Link:

https:/tinygu.mindful

[Barcode hier]

Projektleitung:

Prof. Dr. Sabine Windmann
Psychologisches Institut |Goethe Universität Frankfurt
s.windmann@psych.uni-frankfurt.de | Tel. 069798 35313

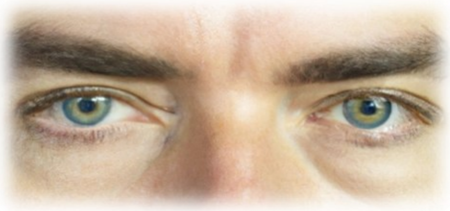

„Mindful“ Waste Separation

A Psychological Intervention

Dear resident,

We are conducting an initiative in Kalbach-Riedberg.

You have been randomly selected to take part in one of the project groups. In this group, we aim to improve the separation of organic waste. We would like to encourage you to handle organic waste **mindfully.**

We also ask you to participate in a survey that focuses specifically on **this topic** (the separation of organic waste in the household).

Here is what you can do:

Collect organic waste through **“mindful” waste separation**:

- ✓ Stick the eyes onto the lid of your **residual** waste bin (e.g., in the kitchen) or place them clearly visible nearby (e.g., countertop, drawer, cabinet). The eyes are intended to increase your **mindfulness**: **No organic waste in the residual** waste bin!
- ✓ Attach the tag to your **black residual** waste bin **outdoors for 2 weeks** (e.g., on the handle of the lid), where you usually dispose of your residual waste.
- ✓ Take part in our short online survey.

Further information: Participation in the project is completely voluntary. You may keep the stickers and tags free of charge in any case. The survey takes approximately 2 minutes, and you can win one of three vouchers worth €100! Further information is available via the barcode or at the link: https:/tinygu.mindful

Projekt Director:

Prof. Dr. Sabine Windmann
Psychologisches Institut |Goethe Universität Frankfurt
s.windmann@psych.uni-frankfurt.de | Tel. 069798 35313

[Barcode hier]

Flyer (Original): Control Group KG

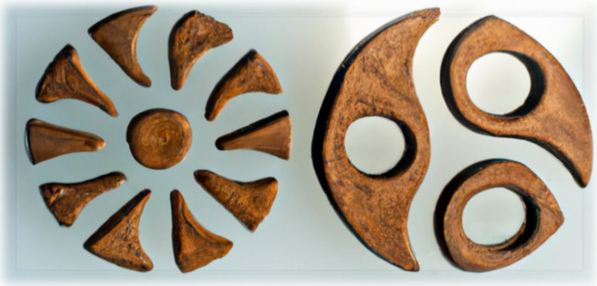

„Mindful“ Wellbeing

Eine Psychologische Intervention

Sehr geehrte Anwohnerin, sehr geehrter Anwohner,

wir führen ein Projekt durch in Kalbach-Riedberg.

Sie sind per Zufall ausgewählt worden, in einer Gruppe des Projekts mitwirken zu können. In dieser Gruppe möchten wir Ihnen **alles Gute** wünschen und Sie ermuntern, **achtsam** mit Ihrer Gesundheit und Ihrem Wohlbefinden umzugehen.

Wir bitten Sie zusätzlich, an einer Befragung teilzunehmen, die einen ganz anderen, **praktischen** Fokus hat (Trennung von Biomüll im Haushalt).

Hier ist, was Sie tun können:

Stärken Sie Ihr Wohlergehen durch „Mindful Wellbeing“:

- ✓ Kleben Sie die Symbole gut sichtbar in Ihrer **Küche** auf eine Kachel oder Schranktür. Sie sollen Ihnen täglich **Gesundheit** und **alles Gute** wünschen.
- ✓ Befestigen Sie das Schildchen an Ihrem **Fahrrad, Ihrer Haustür** oder legen Sie es in ihren **PKW**. Es soll Sie an **Achtsamkeit** im Straßenverkehr erinnern.
- ✓ Nehmen Sie online an unserer Kurzumfrage teil.

Weitere Information: Die Teilnahme an dem Projekt ist vollkommen **freiwillig**. Aufkleber und Anhänger können Sie auf jeden Fall kostenfrei behalten. Die Befragung dauert etwa **2 Minuten** und Sie können einen von drei Gutscheinen über 100 Euro gewinnen! Weitere Informationen erhalten Sie mit dem Barcode oder unter dem Link: https:/tinygu.mindful

Projektleitung:
Prof. Dr. Sabine Windmann
Psychologisches Institut |Goethe Universität Frankfurt
s.windmann@psych.uni-frankfurt.de | Tel. 069798 35313

[Barcode hier]

Flyer (Translation):
Control Group KG

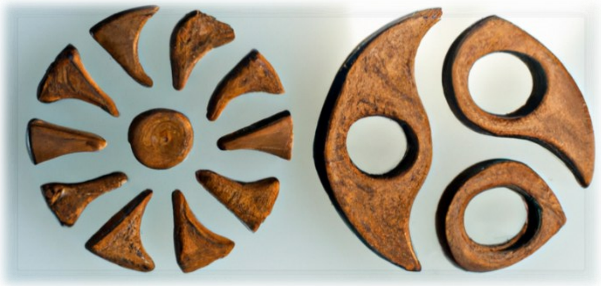

„Mindful“ Wellbeing

A Psychological Intervention

Dear resident,

We are conducting a project in Kalbach-Riedberg.

You have been randomly selected to take part in one of the project groups. In this group, we would like to wish you **all the best** and encourage you to be **mindful** of your health and well-being.

We also ask you to participate in a survey that has a different, **practical focus** (the separation of organic waste in the household).

Here is what you can do:

Stärken Sie Ihr Wohlergehen durch „Mindful Wellbeing“:

- ✓ Place the symbols in a clearly visible spot in your **kitchen** (e.g., on a tile or a cabinet door). They are meant to wish you **health** and **well-being** each day.
- ✓ Attach the tag to your **bicycle**, your **front door**, or place it in your **car**. It is intended to remind you to be **mindful** in traffic.
- ✓ Nehmen Sie online an unserer Kurzumfrage teil.

Further information: Participation in the project is completely voluntary. You may keep the stickers and tags free of charge in any case. The survey takes approximately 2 minutes, and you can win one of three vouchers worth €100! Further information is available via the barcode or at the link: https:/tinygu.mindful

Projektleitung:
Prof. Dr. Sabine Windmann
Psychologisches Institut |Goethe Universität Frankfurt
s.windmann@psych.uni-frankfurt.de | Tel. 069798 35313

[Barcode hier]

<u>Screenshot of the short habit questionnaire as presented in the study (for English translation see main text Figure 3)</u>

**Bitte kreuzen Sie an:**<br><br>**Biomülltrennung ist etwas**	selten oder sehr selten	ziemlich selten	manchmal	ziemlich oft	oft oder sehr oft
... das ich automatisch tue	○	○	○	○	○
... das ich tue, ohne mich bewusst daran erinnern zu müssen	○	○	○	○	○
... das ich tue, ohne nachzudenken	○	○	○	○	○
... das ich anfange zu tun, ohne es zu realisieren	○	○	○	○	○
... Check: kreuzen sie hier bitte einfach „selten oder sehr selten" an	○	○	○	○	○
